# MHC class II deficiency in the Dene native population: a case report highlighting pitfalls in diagnosis and treatment

**DOI:** 10.1186/1710-1492-10-S1-A1

**Published:** 2014-03-03

**Authors:** Alex Lyttle, Chaim Roifman, Harjit Dadi, Nicola Wright, Fotini Kavadas

**Affiliations:** 1 Alberta Children’s Hospital, Calgary, Alberta, Canada; 2 The Hospital for Sick Children, Toronto, Ontario, Canada

## Background

Bare lymphocyte syndrome (BLS) is a rare, hereditary immunodeficiency characterized by the absence of major histocompatibility complex (MHC) class II leading to a form of severe combined immunodeficiency (SCID). Here we present a case introducing BLS in a novel population and emphasize some of the difficulties faced in diagnosing and treating this condition.

## Case

Our patient was a male of Dene native background from the Northwest Territories of Canada. Between the ages of 2 weeks to 6 months of age he had multiple bouts of both viral and bacterial pneumonia, two episodes of bacteremia, and showed poor growth. Despite a low lymphocyte count of 0.9 x 10^9^ cells/L and undetectable immunoglobulins, an immunologist was not initially consulted.

Finally, after his fifth episode of pneumonia he was referred to the Immunology Department in Edmonton and then transferred to the Alberta Children’s Hospital in Calgary. Initial testing revealed IgG levels < 2 g/L, undetectable IgM/IgA and poor response to the diphtheria and tetanus vaccines. Flow cytometry revealed low CD3, CD4 and CD19 counts but normal NK and CD8 counts. Thymidine mitogen testing for T-cell function was low however T-cell receptor excision circle (TREC) analysis was normal. An open thymic biopsy showed evidence of CD4+ maturation arrest. Based on this, MHC class II expression was investigated looking for HLA-DR proteins by flow cytometry and were found to be absent.

A suitable bone marrow donor could not be found. Due to the patient’s deteriorating course, a high resolution hematopoietic stem cell transplantation using a 5/6 umbilical cord donor was performed. Unfortunately, engraftment failed and the child passed away at 19 months of age secondary to respiratory failure.

The genetic testing came back posthumously showing MHC class II deficiency with a RFX5 gene mutation (R400X (1198 c>t).

## Discussion

BLS is extremely rare with less than 200 reported cases worldwide [1] and can result from mutations in one of four regulatory proteins necessary for MHC-II production: CIITA, RFX5, RFXANK and RFXAP [2]. This condition leads to severe CD4+ T-cell dysfunction and thus recurrent bacterial, viral and protozoal infections. Recently TREC studies have begun to be used as part of newborn screening programs for SCID. While sensitive for nearly all forms of SCID [3,4], TREC studies may be normal in BLS [5].

Our case is the first of BLS identified in the Dene native population. Given that early BMT has been shown to have better success rates in treating BLS [6], a screening program may be beneficial in this population.
